# Machine learning-based MRI radiomics for assessing the level of tumor infiltrating lymphocytes in oral tongue squamous cell carcinoma: a pilot study

**DOI:** 10.1186/s12880-024-01210-x

**Published:** 2024-02-05

**Authors:** Jiliang Ren, Gongxin Yang, Yang Song, Chunye Zhang, Ying Yuan

**Affiliations:** 1 Department of Radiology, Shanghai Ninth People’s Hospital, Shanghai Jiao Tong University School of Medicine, No.639 Zhizaoju Road, 200010 Shanghai, China; 2 MR Scientific Marketing, Siemens Healthineers Ltd, 200126 Shanghai, China; 3 Department of Oral Pathology, Shanghai Ninth People’s Hospital, Shanghai Jiao Tong University School of Medicine, No.639 Zhizaoju Road, 200010 Shanghai, China

**Keywords:** Head and neck cancer, Tumor-infiltrating lymphocytes, Magnetic resonance imaging, Machine learning, Radiomics

## Abstract

**Background:**

To investigate the value of machine learning (ML)-based magnetic resonance imaging (MRI) radiomics in assessing tumor-infiltrating lymphocyte (TIL) levels in patients with oral tongue squamous cell carcinoma (OTSCC).

**Methods:**

The study included 68 patients with pathologically diagnosed OTSCC (30 with high TILs and 38 with low TILs) who underwent pretreatment MRI. Based on the regions of interest encompassing the entire tumor, a total of 750 radiomics features were extracted from T2-weighted (T2WI) and contrast-enhanced T1-weighted (ceT1WI) imaging. To reduce dimensionality, reproducibility analysis by two radiologists and collinearity analysis were performed. The top six features were selected from each sequence alone, as well as their combination, using the minimum-redundancy maximum-relevance algorithm. Random forest, logistic regression, and support vector machine models were used to predict TIL levels in OTSCC, and 10-fold cross-validation was employed to assess the performance of the classifiers.

**Results:**

Based on the features selected from each sequence alone, the ceT1WI models outperformed the T2WI models, with a maximum area under the curve (AUC) of 0.820 versus 0.754. When combining the two sequences, the optimal features consisted of one T2WI and five ceT1WI features, all of which exhibited significant differences between patients with low and high TILs (all *P* < 0.05). The logistic regression model constructed using these features demonstrated the best predictive performance, with an AUC of 0.846 and an accuracy of 80.9%.

**Conclusions:**

ML-based T2WI and ceT1WI radiomics can serve as valuable tools for determining the level of TILs in patients with OTSCC.

**Supplementary Information:**

The online version contains supplementary material available at 10.1186/s12880-024-01210-x.

## Introduction

In the head and neck region, oral tongue squamous cell carcinoma (OTSCC) ranks among the most common cancers [1, 2]. In oncology, cancer progression has been associated with the immune response, and tumor evasion from the immune response is a hallmark of cancer [3]. The immune signature, particularly the level of tumor-infiltrating lymphocytes (TILs), plays a key prognostic role in different types of cancers [4, 5]. As part of the complex tumor microenvironment, the TIL level can have a major impact on tumor progression and treatment response [6]. In clinical practice, the TIL level is an established independent prognostic factor for head and neck cancer [7]. However, the spatial and temporal heterogeneity of tumors makes routine pathological assessment of TIL levels difficult. Therefore, accurate and noninvasive determination of TIL levels before treatment remains a challenge for patients with OTSCC.

Magnetic resonance imaging (MRI) is frequently utilized as part of the preoperative evaluation for oral cancers, demonstrating satisfactory diagnostic accuracy when compared to biopsy [8, 9]. Conventional MRI offers high-quality images of soft tissues and plays a significant role in determining the origin, location, and boundaries of oral cancers [10]. Functional MRI techniques, such as diffusion weighted imaging and dynamic contrast-enhanced MRI, allow for the evaluation of microscopic histological features and microvascular parameters, which are crucial in guiding treatment and predicting the prognosis of oral cancers [11]. However, conventional MRI sequences such as T2-weighted (T2WI) and contrast-enhanced T1-weighted (ceT1WI) imaging only provide limited morphological features, which are limited by the detection of human eyes. In recent years, radiomics has expanded the capabilities of routine medical imaging in clinical oncology. Radiomics involves extracting high-throughput features from medical images to facilitate oncology predictions [12]. Conventional MRI radiomics has been proven to be useful in predicting histopathological characteristics, stage, treatment response, and survival in patients with head and neck cancer [13–17]. Moreover, several studies have reported that MRI radiomics can stratify the tumor-immune microenvironment of breast cancer [18], ductal adenocarcinoma [19], and rectal cancer [20]. Meyer et al. [21] reported that histogram features derived from T2WI and ceT1WI might reflect the TIL levels in head and neck cancer. However, their study did not incorporate second- and high-order texture features and transformed domain images, which could have overlooked crucial tumor information.

Therefore, this study aims to explore the performance of machine learning (ML)-based MRI radiomics for predicting TIL levels in patients with OTSCC.

## Materials and methods

### Patient population

This study was approved by the Institutional Review Board of Shanghai Ninth People’s Hospital and informed consent was waived due to retrospective study design. The study retrospectively included consecutive patients with OTSCC who received treatment at our hospital between April 2018 and December 2022. The inclusion criteria were as follows: (1) patients with pathologically diagnosed OTSCC after surgery, (2) patients who underwent standard MRI within 14 days before surgery, and (3) patients with complete relevant clinical data. The study’s exclusion criteria were (1) patients who had undergone any form of treatment before undergoing MRI; (2) lesions deemed too small, with a minimum diameter of < 10 mm; and (3) MRI scans of insufficient quality due to artifacts or subjects’ motion.

### Pathologic analysis

**Fig. 1:**
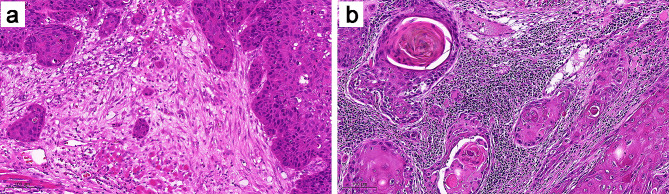
Tumor-infiltrating lymphocytes (TILs) were evaluated on full sections of OTSCC stained with HE. Representative samples classified as low (≤ 60%) stromal TILs **(a)** and high (> 60%) stromal TILs **(b)**

Complete H&E-stained sections obtained from the surgical specimens of all patients were available for analysis. TIL levels in the stromal compartment were evaluated using a scoring method recently introduced by the International Immuno-Oncology Biomarkers Working Group [22]. The presence of fibrosis or central necrosis was not considered when assessing TILs. TIL levels were calculated as the proportion of lymphocytes infiltrated into the stroma, measured in increments of 10%. Patients were categorized into groups based on the median value of TILs (60%), with samples having TIL levels of ≤ 60% considered as those with low TILs and samples with TIL levels of > 60% considered as those with high TILs (Fig. 1).

### Image acquisition and segmentation

MRI examinations were performed using a 3.0-T scanner equipped with a head-and-neck array coil (Ingenia; Philips Healthcare). The imaging protocol included axial fat-suppressed T2WI and ceT1WI. The scanning parameters are presented in Supplementary Table 1. A standard dose of 0.1 mmol/kg of gadopentetate dimeglumine (Magnevist, Bayer, Mullerstrasse, Germany) was administered for ceT1WI.

**Fig. 2:**
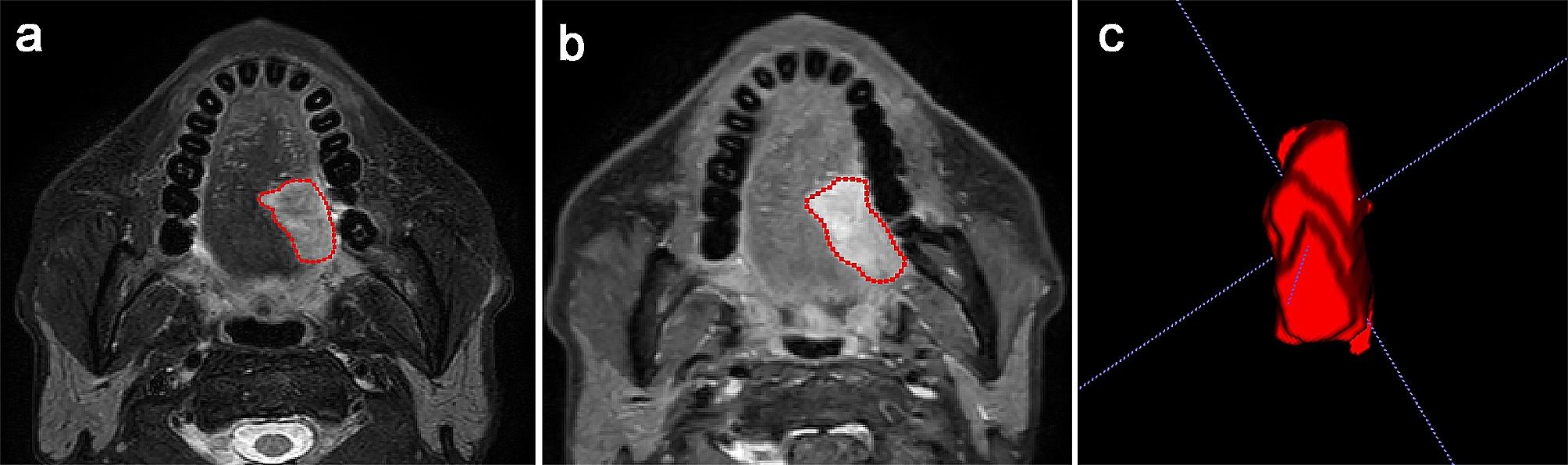
Tumor segmentation in OTSCC. The segmentation was performed on T2WI **(a)** and ce-T1WI **(b)**. The regions of interest **(c)** covering the entire tumor were acquired by stacking all slices with the tumor

ITK-SNAP software (www.itk-snap.org) was used for tumor segmentation. A radiologist with 8 years of experience in interpreting head and neck MRI carefully outlined the regions of interest (ROI) encompassing the entire tumor (Fig. 2). To assess the interobserver variability of the radiomics features in the segmentation process, an additional radiologist with 9 years of experience in head and neck MRI interpretation randomly selected 20 lesions for delineation of ROIs. Both radiologists were blinded to the clinical details and pathological results.

### Image processing and feature extraction

Before extracting radiomics features, three image processing techniques were applied to all images: (i) gray-level normalization by centering it at the mean with standard deviation (scale, 100), (ii) resampling and rescaling of pixels (resultant pixel size, 1 × 1 mm^2^), and (iii) gray-level discretization (bin count, 64).

Radiomics features were extracted using an open-source python package (Pyradiomics, version 3.0.1, www.radiomics.io). A total of 14 shape- and size-based features, 17 first-order histogram features, and 75 textural features were extracted from each sequence. The texture feature was further classified into five distinct classes: gray-level cooccurrence matrix (GLCM, *n* = 24), gray-level run-length matrix (GLRLM, *n* = 16), gray-level size zone matrix (GLSZM, *n* = 16), gray-level dependence matrix (GLDM, *n* = 14), and neighboring gray tone difference matrix (NGTDM, *n* = 5). The Laplacian of Gaussian (LoG) filtering technique was applied to the original images at three different scales (1, 3, and 5 mm), and the wavelet transform was performed on four distinct combinations of high- and low-frequency bands. The processed images were used separately to calculate the histogram and textural features. In total, 750 radiomics features were obtained from each sequence. The algorithms used for acquiring radiomics features were sourced from the image biomarker standardization initiative [23].

### Dimension reduction

The intraclass correlation coefficients (ICCs) were computed for each feature, and the interpretability of reproducibility for each feature was determined using the following scale: (i) ICC < 0.4, indicating poor reproducibility; (ii) 0.59 > ICC ≥ 0.4, indicating fair reproducibility; (iii) 0.75 > ICC ≥ 0.6, indicating good reproducibility; and (iv) ICC ≥ 0.75, indicating excellent reproducibility (20). Radiomics features with ICC value ≥ 0.8 were deemed stable and included in subsequent analyses.

Spearman correlation coefficients (*r*) were employed to detect collinearity among features, with a threshold of 0.8 for *r* considered as indicative of high collinearity. When a pair of features had high collinearity, the one with higher collinearity than the other was excluded.

### Machine learning

Optimal features were selected using the minimum-redundancy maximum-relevance (MRMR) approach, which ranked the features based on their relevance-redundancy scores, measuring mutual information between each feature and the lesion labels, while being compared with the average mutual information with previously selected features. MRMR has the advantage of selecting features with high predictive power while reducing the impact of redundant features on the model. To avoid overfitting of the model, we applied the principle that has been reported in previous studies [13, 24], which states that the maximum number of features should be 1/10 of the number of patients. Therefore, the top six features were respectively chosen using T2WI, ceT1WI alone, and their combination. Additionally, clinical variables were taken into account when selecting features.

Due to the small sample size, three representative and relatively simpler classification models, namely, logistic regression (LR), random forest, and support vector machine (SVM), were constructed based on the optimal subset. LR is suitable for classification of linear data and has strong interpretability. The RF classifier consists of a collection of decision trees, is robust to noises and outliers, and can rapidly handle high-dimension data. Support vector machines are suitable for processing large-scale data sets and solving nonlinear problems. Support vector machines are suitable for processing large-scale data sets and solving nonlinear problems. The models’ performances were evaluated through 10-fold cross-validation.

### Statistical analyses

Statistical analysis was conducted using R software (version 3.5.2; www.r-project.org). Several R packages were used for specific tasks: the “caret” package was employed to calculate the Spearman correlation coefficient, the “mRMRe” package was used to implement the MRMR algorithm, and both the “randomForest” and “e1071” packages were used for ML-based classification. Mann–Whitney U tests were performed to assess the association between radiomics features and TILs. Receiver operating characteristic analysis was conducted for each model, and metrics such as the area under the curve (AUC), accuracy, sensitivity, and specificity were calculated. The DeLong test was used for comparing the performance of classification models. Statistical significance was determined by a *P* value of 0.05 for all tests.

## Results

### Patients

A total of 68 patients with OTSCC were eligible for our study, including 30 patients with high TILs. Patients with high TILs were significantly older than those with low TILs (*P* = 0.012). No significant differences were noted in sex, maximum diameter, tumor. The clinicopathological characteristics of all patients are presented in Table 1.

**Table 1:** Detailed clinicopathological characteristics of the patients

	Low TIL group (*N* = 38)	High TIL group (*N* = 30)	***P*** value
Sex			0.686
Male	22 (57.9%)	15 (50%)	
Female	16 (42.1%)	15 (50%)	
Age (years)	52 (39, 61)	63 (48, 68)	0.012
Maximum diameter (mm)	34 (26, 45)	33 (27, 38)	0.897
Tumor thickness (mm)	14 (9, 18)	11 (8, 18)	0.299
Clinical T stage			0.763
T1-2	19 (50%)	17 (56.7%)	
T3-4	19 (50%)	13 (43.3%)	
Clinical N stage			0.161
N0	33 (86.8%)	21 (70%)	
N1-3	5 (13.2%)	9 (30%)	
Histological grade			0.988
I/I-II	23 (60.5%)	19 (63.3%)	
II/II-III/III	15 (39.5%)	11 (36.7%)	

Data are expressed as median (interquartile range) or number (percentage)

### Dimension reduction

**Fig. 3:**
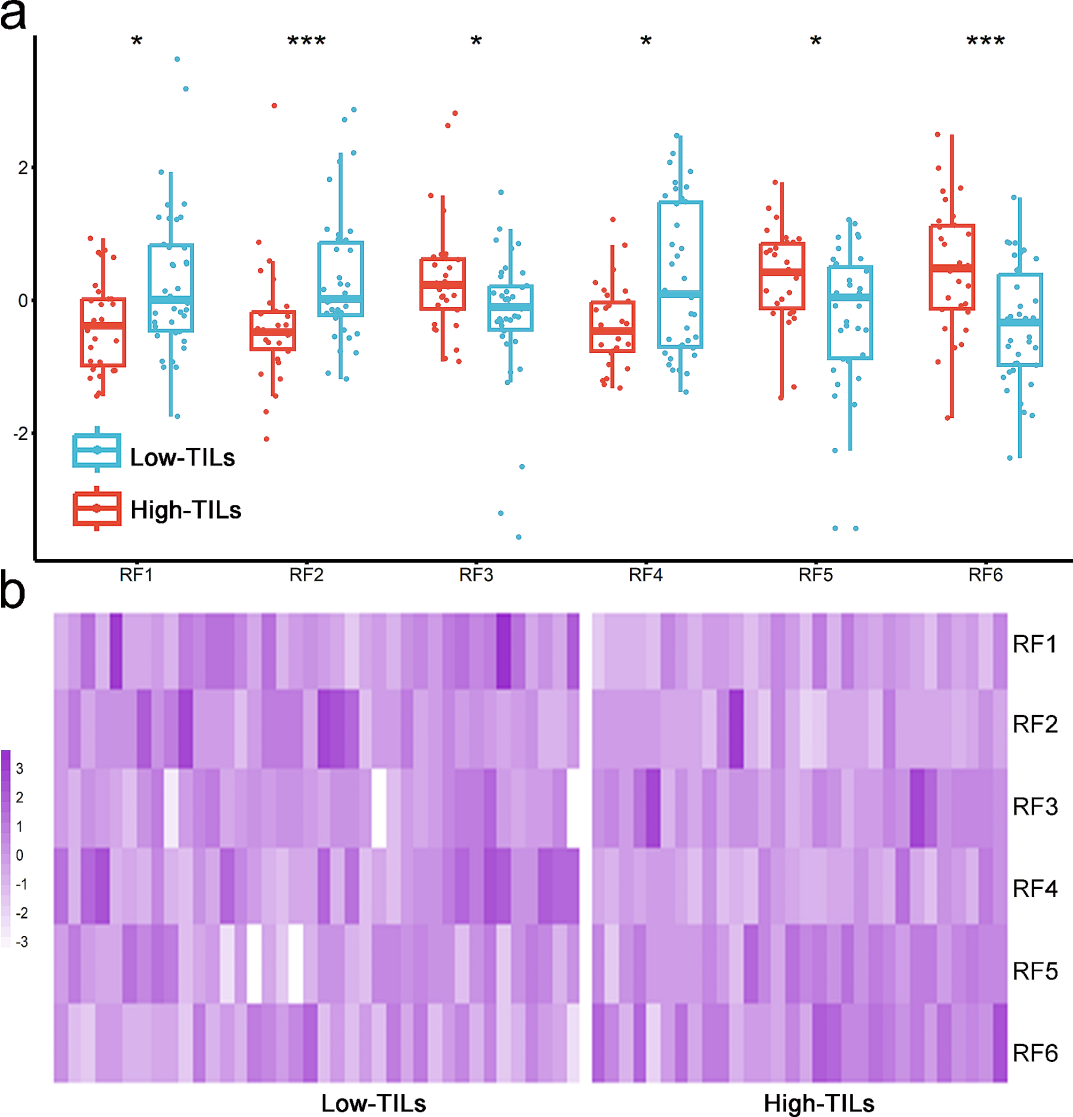
Comparison of the normalized (Z score transformation) features selected from the combined sequences between patients with high and low TILs. Boxplots of the radiomics features between the two groups **(a)** are shown. Significant differences between the two groups were observed in all selected features (^*^P < 0.05, ^**^P < 0.01, ^***^P < 0.001). Colored heatmaps **(b)** show distributions and differences in the radiomics features between the two groups. TILs, tumor-infiltrating lymphocytes; RF, radiomics feature. Please refer to Table 2 for the code name of the selected features

**Fig. 4:**
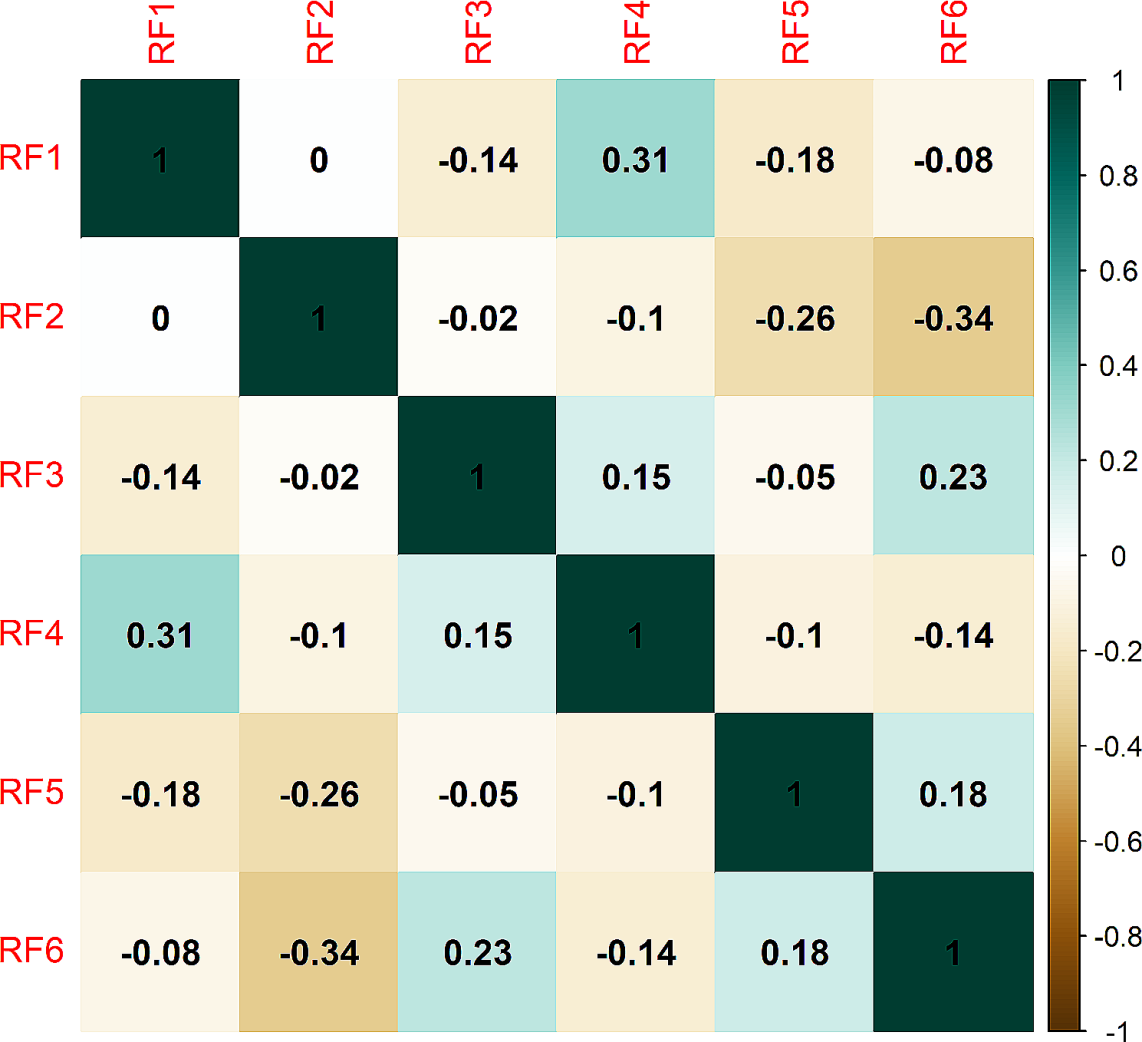
Auto- and cross-correlation of the features selected from the combined sequences are shown as a correlation matrix. Significant cross-correlation (Spearman correlation coefficient > 0.7) was not observed among features. Please refer to Table 2 for the code name of the selected features

The reproducibility analysis conducted by two radiologists revealed that 66.3% (497/750) of T2WI features and 82.8% (621/750) of ceT1WI features exhibited stability, with an ICC value of ≥ 0.8. Subsequently, the elimination of highly collinear features resulted in a reduction in the number of features to 122, including 49 T2WI and 73 ceT1WI features. The top six features selected from T2WI and ceT1WI alone using MRMR are presented in supplementary Table 2. When the two sequences were combined, the selected features consisted of one T2WI and five ceT1WI features, and no clinical characteristics were included. Furthermore, two features were extracted from the original images, as well as from images processed with the LoG filter and wavelet transformation. The classes of optimal features included three histogram features, two GLCM features, and one GLDM feature. All features from the combined sequences showed significant differences between the two groups and had AUC values ranging from 0.650 to 0.751 (Table 2). The normalized values (Z score transformation) of the selected features are displayed in Fig. 3. There was no significant collinearity among the selected features from the combined sequences (Fig. 4).

**Table 2:** Comparison of the radiomics features selected from combined sequences between the two groups and diagnostic performance of the features

Code	Sequence	Image type	Feature class	Feature name	Low TILs	High TILs	***P*** value	AUC (95% CI)
RF1	ceT1WI	LoG_3 mm_	Histogram	Kurtosis	3.04 (2.79, 3.48)	2.84 (2.52, 3.05)	0.011	0.681 (0.554, 0.808)
RF2	ceT1WI	Wavelet_HH_	Histogram	Skewness	0.11 (0.04, 0.34)	−0.02 (− 0.10, 0.06)	< 0.001	0.743 (0.621, 0.865)
RF3	ceT1WI	Original	GLCM	ClusterShade	−1032 (− 2032, − 146)	−110 (− 1154, 986)	0.035	0.650 (0.5317, 0.783)
RF4	ceT1WI	Original	GLDM	DependenceVariance	0.68 (0.50, 1)	0.56 (0.49, 0.66)	0.025	0.660 (0.530, 0.790)
RF5	ceT1WI	LoG_1 mm_	Histogram	Minimum	−115 (− 143, − 101)	−104 (− 121, − 91)	0.016	0.672 (0.543, 0.801)
RF6	T2WI	Wavelet_HL_	GLCM	JointEntropy	8.32 (8.02, 8.68)	8.69 (8.41, 8.99)	< 0.001	0.751 (0.633, 0.868)

AUC: area under the receiver operating characteristic curve; ceT1WI: contrast-enhanced T1-weighted imaging; GLCM: gray-level cooccurrence matrix; GLDM: gray-level dependence matrix; RF: radiomics feature; T2WI: T2-weighted imaging

### Classification performance

**Fig. 5:**
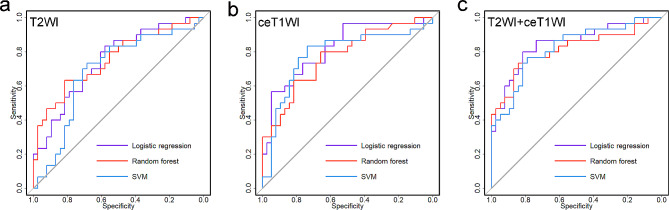
Classification performance of the machine learning models in discriminating between OTSCCs with high and low levels of TILs. Based on the features selected from each sequence alone, the ceT1WI models **(b)** outperformed the T2WI models **(a)**, with a maximum AUC of 0.820 versus 0.754. Upon combining the two sequences **(c)**, the logistic regression model exhibited the best predictive performance, with an AUC of 0.846. SVM, support vector machine

When utilizing T2WI alone, the classification models constructed with LR, random forest, and SVM achieved AUCs of 0.746, 0.754, and 0.688 and accuracies of 69.1%, 73.5%, and 70.6%, respectively, whereas when utilizing ceT1WI alone, the classification models achieved AUCs of 0.820, 0.771, and 0.782 and accuracies of 77.9%, 72.1%, and 77.9%, respectively. Based on the features from the combined sequences, LR demonstrated the best performance in predicting the TILs level in OTSCCs, yielding an AUC of 0.846 and an accuracy of 80.9%. In comparison, random forest and SVM showed worse performance with AUCs of 0.813 and 0.822 and accuracies of 79.4% and 77.9%, respectively, although significant differences were not observed in the DeLong tests (*P* = 0.447 and 0.451). The predictive performances of these classification models are presented in Table 3; Fig. 5.

**Table 3:** Performance of the machine learning-based classifications

	AUC	Accuracy	Sensitivity	Specificity
T2WI				
Logistic regression	0.746 (0.630, 0.863)	69.1 (68.5, 69.7)	83.3 (70.0, 96.7)	57.9 (42.2, 73.6)
Random forest	0.754 (0.635, 0.872)	73.5 (73.0, 74.1)	63.3 (46.1, 80.6)	81.6 (69.3, 93.9)
SVM	0.688 (0.556, 0.820)	70.6 (70.0, 71.2)	73.3 (57.5, 89.2)	68.4 (53.6, 83.2)
ceT1WI				
Logistic regression	0.820 (0.718, 0.922)	77.9 (77.4,78.4)	56.7 (38.9, 74.4)	94.7 (87.6, 100)
Random forest	0.771 (0.659, 0.884)	72.1 (71.5,72.6)	80.0 (65.7, 94.3)	65.8 (50.7, 80.9)
SVM	0.782 (0.661, 0.902)	77.9 (77.4, 78.4)	83.3 (70.0, 96.7)	73.7 (59.7, 87.7)
T2WI + ceT1WI				
Logistic regression	0.846 (0.750, 0.943)	80.9 (80.4, 81.3)	80.0 (65.7, 94.3)	81.6 (69.3, 93.9)
Random forest	0.813 (0.703, 0.924)	79.4 (78.9, 79.9)	73.3 (57.5, 89.2)	84.2 (72.6, 95.8)
SVM	0.822 (0.721, 0.923)	77.9 (77.4, 78.4)	76.7 (61.5, 91.8)	78.9 (66.0, 91.9)

Data are presented as percentages except AUC; 95% CIs are included in parentheses

AUC: area under the curve; CI: confidence interval; SVM: support vector machine

## Discussion

Our preliminary study showed that the logistic regression classifier combining T2WI and ceT1WI radiomics features can achieve satisfactory performance in predicting TIL levels in patients with OTSCC.

Radiomic features have the ability to identify tissue characteristics and pathological properties by analyzing the spatial distribution of pixel intensity within a tumor [25, 26]. Previous studies have confirmed correlations between T2WI and T1WI histogram parameters and TIL levels in head and neck cancer [21] and between MRI radiomics signatures and TIL levels in breast cancer [27]. In our study, the selected features from the combined sequences showed significant differences between the groups and achieved AUCs ranging from 0.650 to 0.751 in predicting the TIL levels in patients with OTSCC. These features mainly consisted of two classes: histogram and GLCM. The histogram features analyze the gray-levels of individual pixels without taking into account the context of surrounding pixels, whereas features derived from GLCM describe the interactions and correlations between pairs of pixels. The combination of two classes of features characterizes image texture at different scales and provides more informative and discriminative information. Interestingly, the optimal subsets from the combined sequences included more features derived from ceT1WI (*n* = 5) than those derived from T2WI (*n* = 1). Moreover, when constructing predictive models using each sequence alone, ceT1WI demonstrated better performance than T2WI (maximum AUC: 0.820 vs. 0.754). These findings suggest that the heterogeneity of blood supply, as reflected by ceT1WI features, has stronger associations with TILs than the water content, as reflected by T2WI features. Notably, the predictive model built by combining the two sequences achieved superior performance (maximum AUC: 0.846) compared with that built using each sequence alone. This finding is consistent with previous studies showing the advantage of radiomics analysis combined with multiparametric MRI in predicting lymph node metastasis [13], HPV status [28], and histological grade [29] in head and neck cancers. These results further highlight the potential of radiomics analysis based on different MRI sequences to provide supplemental information for oncology prediction. However, this study only considered conventional MRI for the analysis. A previous study by Meyer et al. [30] illustrated that parameters obtained from dynamic-contrast-enhanced MRI can reflect the expression of TILs in head and neck cancer. Therefore, investigating the integration of radiomics analysis, combining both conventional and functional MRI, is imperative for predicting TIL levels, specifically in cases of OTSCC.

In radiomics analysis, a large number of features are extracted from medical images to build predictive models. However, in our study, we only analyzed the MRI of 68 carefully selected OTSCCs. It is important to acknowledge that a limited sample size can impact the stability and reproducibility of the results. Firstly, insufficient sample data can introduce random errors in feature extraction. Secondly, it can lead to overfitting and affect the model’s generalization ability. Finally, small sample sizes may not allow for the detection of subtle but important effects. As an exploratory study, we followed a rigorous radiomics analysis process that included image preprocessing, inter-observer variability analysis, and more. This approach aimed to minimize the impact of small sample sizes on result stability. Additionally, we employed a ten-fold cross-validation method to evaluate our model’s performance. In this process, all samples were used for both training and validation, enabling us to assess its stability. To explore the relationship between clinical variables and TILs, as well as the independent value of radiomics in predicting TILs, clinical variables were included in feature selection. In feature selection, we found that no clinical variables were selected, which indirectly highlights the advantages of radiomics in the preoperative evaluation of TILs. Regarding model construction, determining the most efficient ML algorithm becomes particularly valuable for this small sample research. To address this challenge, we compared the discriminatory capabilities of three different classifiers. Remarkably, we found that logistic regression outperformed random forest and SVM (maximum AUCs: 0.846 vs. 0.813 and 0.822). Our data may be linear or linearly separable, making the LR model using a linear algorithm more effective than nonlinear approaches such as random forest and SVM. Furthermore, when dealing with small sample cohorts, LR, being a simple algorithm, tends to exhibit better stability than more complex algorithms. As indicated previously, many studies have shown that head and neck cancers with elevated TIL level often exhibit improved biological behavior and are associated with enhanced overall and disease-free survival rates [31, 32]. TIL level has become a prognostic factor for head and neck cancer independent from TNM staging. While this study lacks external validation, it represents a novel and preliminary attempt to noninvasively assess TIL level in OTSCC using conventional MRI radiomics. Based on our research findings, patients are expected to achieve more precise risk stratification prior to surgery, leading to more personalized treatment decisions.

Our study had three main limitations. First, it was a retrospective, single-center study with a small sample. Sample selection bias could not be eliminated due to the strict enrollment criteria utilized. The value of the radiomics model necessitates its validation through multicenter and large-scale studies. Second, we focused only on conventional MRI in our study. However, we believe that exploring the potential of radiomics based on functional sequences, such as diffusion-weighted imaging and dynamic-contrast-enhanced MRI, would be highly beneficial. Third, many studies have reported that radiomics features extracted from the peritumoral regions can provide additional information to the intratumor regions for predicting histopathological features in breast cancer [33], lung cancer [34], and gliomas [35]. The value of radiomics information from the peri-tumor region for assessing TILs in OTSCCs deserves to be further explored. Finally, the process of manually delineating all ROIs is time-consuming and prone to variation between different observers. Hence, developing an automatic segmentation approach in the future is imperative.

## Conclusions

In summary, our study provided preliminary evidence supporting the effectiveness of an ML-based MRI radiomics approach in predicting the level of TILs in OTSCC. The LR model based on the combined sequences demonstrated the best diagnostic performance and may facilitate clinical decision-making for patients with OTSCC.

### Electronic supplementary material

Below is the link to the electronic supplementary material.

Supplementary Material 1

## Data Availability

The datasets used and/or analyzed during the current study are available from the corresponding author on reasonable request.

## Abbreviations

AUC: Area under the curve
ceT1WI: Contrast-enhanced T1-weighted imaging
ICC: Intraclass correlation coefficient
LoG: Laplacian of Gaussian
LR: Logistic regression
MRI: Magnetic resonance imaging
ROC: Receiver operating characteristic
ROI: Region of interest
SVM: Support vector machine
OTSCC: Oral tongue squamous cell carcinoma
T2WI: T2-weighted imaging
TE: Echo time
TR: Repetition time
TIL: Tumor-infiltrating lymphocyte
