# Supplementary material for: Machine learning-based MRI radiomics for assessing the level of tumor infiltrating lymphocytes in oral tongue squamous cell carcinoma: a pilot study
Source: BMC Med Imaging. 2024 Feb 5;24:33. doi: 10.1186/s12880-024-01210-x (PMC10845803; doi:10.1186/s12880-024-01210-x)
Supplement: Supplementary file 1 — Supplementary Material 1 [file 12880_2024_1210_MOESM1_ESM.docx]

**Supplementary Table 1** The scanning parameters

| Sequence | TR | TE | Matrix | Field of view | Slice thickness | Slice gap |
| --- | --- | --- | --- | --- | --- | --- |
| T2WI | 2800 ms | 85 ms | 256 × 192 | 240 mm × 240 mm | 3mm | 1mm |
| ceT1WI | 580 ms | 15 ms | 256 × 192 | 240 mm × 240 mm | 3mm | 1mm |

ceT1WI, contrast-enhanced T1-weighted imaging; T2WI, T2-weighted imaging; TE Echo time; TR Repetition time.

**Supplementary Table 2** The radiomics features selected from each sequence alone

| Sequence | Image type | Feature class | Feature name |
| --- | --- | --- | --- |
| T2WI | Original | Histogram | Kurtosis |
|  | Wavelet_HH_ | Histogram | Skewness |
|  | Wavelet_LH_ | GLCM | Correlation |
|  | LoG_5mm_ | GLRLM | ShortRunHighGrayLevelEmphasis |
|  | LoG_3mm_ | NGTDM | Busyness |
|  | Wavelet_HL_ | GLCM | JointEntropy |
| ceT1WI | Original | GLSZM | LargeAreaHighGrayLevelEmphasis |
|  | LoG_3mm_ | Histogram | Kurtosis |
|  | Wavelet_HL_ | Histogram | Minimum |
|  | Original | GLCM | ClusterShade |
|  | Wavelet_HH_ | Histogram | Skewness |
|  | Wavelet_HL_ | GLCM | JointEntropy |

GLCM, gray-level cooccurrence matrix; GLRLM gray-level run-length matrix; GLSZM gray-level size zone matrix; NGTDM neighboring gray tone difference matrix.
